# Network-based integrative analysis of multi-level regulatory mechanisms associated with glyphosate exposure

**DOI:** 10.3389/fbinf.2026.1890219

**Published:** 2026-08-20

**Authors:** Claudia Consuegra-Mayor, Barbara Arroyo-Salgado, Jesus Olivero-Verbel

**Affiliations:** 1 Environmental and Computational Chemistry Group, School of Pharmaceutical Sciences, Zaragocilla Campus, University of Cartagena, Cartagena, Colombia; 2 Biomedical, Toxicological and Environmental Sciences Group, School of Medicine, Zaragocilla Campus, University of Cartagena, Cartagena, Colombia; 3 School of Health Sciences, University of San Buenaventura, Cartagena, Colombia

**Keywords:** biomarkers, epigenetic regulation, hub genes, network biology, transcriptional regulation

## Abstract

Understanding the molecular effects associated with glyphosate exposure remains challenging due to the fragmentation of available evidence across heterogeneous data sources. This study aimed to integrate heterogeneous molecular evidence related to glyphosate exposure through a reproducible systems biology workflow in order to prioritize human genes, regulatory networks, and biological processes associated with glyphosate. Multiple platforms, including the Comparative Toxicogenomics Database (CTD), GeneShot, and GeneCards, were queried and complemented with artificial intelligence-assisted information retrieval. Genes present in at least two independent sources were selected, and additional candidates were obtained from transcriptomic datasets using GEO2R. Gene identifiers were standardized according to the HUGO Gene Nomenclature Committee (HGNC). Functional enrichment and protein-protein interaction (PPI) network analyses were performed using STRING and Cytoscape, and hub genes were identified using the cytoHubba plugin. In addition, upstream transcription factor analysis was conducted to identify potential regulatory drivers of the gene network. The resulting consensus dataset was subsequently analyzed using protein-protein interaction networks, functional enrichment, and upstream regulatory inference. The integrative workflow prioritized a core set of 50 genes was identified, with key hub genes including *TP53, BCL2, IL6, CASP3, ALB*, and *TNF*. Enrichment analyses revealed a consistent overrepresentation of pathways related to cellular stress response, apoptosis, endocrine signaling, and cancer, along with a specific epigenetic signal associated with DNA methylation. Upstream regulatory analysis identified key transcription factors linked to hormonal signaling, cellular stress response, and transcriptional control, further supporting the hierarchical organization of the gene network. Gene-disease and phenotype associations further highlighted links with hepatobiliary disorders, neoplastic processes, and endocrine alterations. Overall, this integrative systems biology framework provides a comprehensive view of the molecular architecture associated with glyphosate exposure, prioritizing candidate genes, regulatory networks, and biological processes for hypothesis generation and future experimental and epidemiological validation.

## Highlights

- An integrative network-based workflow prioritized 50 glyphosate-associated genes by integrating multiple independent molecular data sources.
- Protein-protein interaction analysis identified *TP53, BCL2, IL6, CASP3, ALB*, and *TNF* as central hub genes within a highly interconnected molecular network.
- Functional enrichment analyses revealed coordinated molecular responses involving cellular stress, apoptosis, endocrine signaling, and cancer-related pathways.
- Upstream regulatory analysis identified transcription factors associated with hormonal signaling, cellular stress responses, differentiation, and transcriptional regulation. Gene-disease and phenotype enrichment associated the prioritized molecular network with hepatobiliary disorders, neoplastic processes, and endocrine alterations.
- The integrative workflow provides a reproducible framework for molecular prioritization and hypothesis generation to support future experimental and epidemiological studies.

## 1 Introduction

Glyphosate is a broad-spectrum herbicide whose use is widespread worldwide in agriculture, particularly in genetically modified crops that are resistant to its action (Benbrook, 2016; Clements et al., 2017; Connolly et al., 2020; Soukup et al., 2020). Its mechanism of action is based on the inhibition of the enzyme five-enolpyruvylshikimate-3-phosphate synthase (EPSPS), which is essential for the synthesis of aromatic amino acids in the shikimate pathway, present in plants, fungi, and bacteria (Boocock and Coggins, 1983). Enzymatic inhibition causes dysregulation of the metabolic pathway, generating an uncontrolled carbon flux that leads to sustained accumulation of shikimic acid in the plant. This metabolic imbalance results in a progressive deterioration of its functionality and viability (Cerdeira and Duke, 2006). Since this pathway does not exist in animals, glyphosate was long assumed to have low toxicity for mammals (European Food Safety Authority (EFSA), 2017; Solomon et al., 2007; Williams et al., 2012). However, the accumulated scientific evidence has challenged this premise, demonstrating that exposure, especially when chronic or at high concentrations, can induce adverse effects in higher organisms, including humans (Myers et al., 2016; Torretta et al., 2018).

Various experimental and epidemiological studies have reported that exposure to glyphosate can cause alterations in multiple organs and systems. The main mechanism responsible for these effects is oxidative stress, which leads to an imbalance between oxidant and antioxidant systems, causing direct damage to lipids, proteins, and DNA, and leading to cell death via apoptosis (Wang et al., 2022). This herbicide has been linked to multiple cellular processes and pathologies in specific organs. In the liver, manifestations such as fibrosis and steatosis have been reported, suggesting that glyphosate may contribute to the development of non-alcoholic fatty liver disease (Mesnage et al., 2015; Ren et al., 2019). At the renal level, glomerulopathies, mitochondrial damage, and alterations in renal function have been described, possibly associated with the dose or duration of exposure (Mesnage et al., 2015; Zhang et al., 2017).

The endocrine system is also affected, with reports of hormonal disruption (Mohammadi et al., 2022) and adverse effects on fertility and fetal development (Avdatek et al., 2018; Ingaramo et al., 2016). These alterations have been linked, in part, to glyphosate’s potential to act as an endocrine disruptor (Ingaramo et al., 2016; Defarge et al., 2016; Geier and Geier, 2023; Warner et al., 2020).

Furthermore, some studies have suggested an association between glyphosate exposure and an increased risk of neoplastic diseases, particularly non-Hodgkin lymphoma (Portier, 2020; Zhang et al., 2019; Weisenburger, 2021). However, the results remain controversial and inconclusive, partly due to methodological differences, the experimental models used, and the lack of systematic integration of available data (Singh et al., 2024). Exposure to glyphosate has also been linked to an increased risk of chronic diseases such as diabetes, asthma, osteoporosis, obesity, and neurodegenerative diseases, including Alzheimer’s and Parkinson’s disease (Mesnage and Antoniou, 2017).

From a molecular perspective, glyphosate has been reported to induce DNA damage (Kaur and Kaur, 2018; Poletta et al., 2009; Woźniak et al., 2018) through multiple mechanisms, including the generation of reactive oxygen species (ROS) (Martínez et al., 2020), DNA strand breaks (Kwiatkowska et al., 2017), and micronucleus formation (De Castilhos Ghisi et al., 2016; Nagy et al., 2021). In parallel, experimental evidence has shown that environmentally relevant concentrations of glyphosate can promote proliferation in hormone-dependent breast cancer cells through activation of estrogen receptor α (ERα) and modulation of estrogen response elements (EREs), effects that may be reversed by receptor antagonists (Mesnage et al., 2017a; Thongprakaisang et al., 2013; Muñoz et al., 2023). Additionally, ligand-independent signaling pathways involving cAMP/PKA-mediated ERα activation have also been proposed, suggesting a disruption in the balance between proliferative and apoptotic responses (Mesnage et al., 2017a). These alterations compromise genomic integrity and may promote carcinogenesis. Moreover, effects on the epigenome have been identified, notably changes in DNA methylation patterns, alterations in the expression of non-coding RNAs, and even modifications in chromatin structure (Warner et al., 2020; Kubsad et al., 2019; Marko et al., 2009). Such epigenetic changes are significant not only because of their potential reversibility but also due to their involvement in gene regulation, adaptive responses, and transgenerational effects (Kubsad et al., 2019).

A critical limitation in understanding the biological effects of glyphosate lies not only in the fragmentation of the available molecular evidence in the absence of systematic strategies to integrate heterogeneous datasets into a coherent biological framework. Although numerous studies have reported genes associated with glyphosate exposure across different experimental models and data repositories (De Souza et al., 2019; Larsen et al., 2022; Mesnage et al., 2021; Woźniak et al., 2021), these findings remain largely isolated, limiting the identification of consistent molecular signatures, functional hierarchies, and regulatory interactions. Consequently, there is a growing need for reproducible systems biology approaches capable of integrating complementary sources of evidence to support robust molecular prioritization and biological interpretation (Masseroli et al., 2014; Merelli et al., 2014). In this context and given the complexity of the molecular and cellular effects associated with glyphosate, the use of bioinformatics tools has gained strength in recent years as a complementary strategy in toxicological assessment (Miryala et al., 2018).

Network-based biological interaction analyses provide a systems-level framework for exploring functional relationships among genes and proteins through protein-protein interaction (PPI) networks, where nodes represent proteins and edges correspond to experimentally supported or computationally predicted interactions (Hawe et al., 2019; Milano and Cannataro, 2023; Milano et al., 2018). Combined with functional enrichment analyses based on resources such as Gene Ontology (GO), KEGG, and Human Phenotype Ontology (HPO), these approaches enable the biological contextualization of prioritized gene sets, facilitating the identification of coordinated pathways, regulatory processes, and disease-associated molecular signatures (The Gene Ontology Consortium, 2015; Kanehisa et al., 2016). Owing to the increasing availability of genomic resources, these methodologies have become fundamental tools for interpreting complex molecular datasets and generating biologically meaningful hypotheses in systems toxicology (Miryala et al., 2018; Joshi et al., 2024).

The present study implements a two-stage integrative systems biology workflow that combines molecular evidence retrieval from multiple complementary resources with transcriptomic information to generate a consensus set of glyphosate-associated genes using predefined selection criteria. This prioritized gene set was subsequently characterized through protein–protein interaction network analysis, functional enrichment, and upstream regulatory inference to identify coordinated molecular processes and regulatory relationships. By integrating heterogeneous molecular evidence into a unified analytical framework, this approach provides a systems-level perspective of biological architecture associated with glyphosate exposure and generates prioritized candidates for future experimental and epidemiological investigation.

## 2 Materials and methods

### 2.1 Genomic information retrieval

The identification of genes associated with glyphosate exposure was performed using a data mining strategy integrating multiple bioinformatics platforms and literature-based resources. The sources consulted included the Comparative Toxicogenomics Database (CTD; http://ctdbase.org/), the GeneShot platform (https://maayanlab.cloud/geneshot/), the GeneCards database (http://www.genecards.org/), and an AI-assisted literature retrieval approach supported by ChatGPT (OpenAI; https://chatgpt.com/). The search term “Genes and Glyphosate” was applied across all platforms to identify human genes potentially associated with glyphosate exposure. All databases and online resources were accessed in April 2025.

### 2.2 Gene identification and selection

#### 2.2.1 Comparative toxicogenomics database (CTD)

Gene–chemical interaction data were retrieved from the Comparative Toxicogenomics Database (CTD) (Davis et al., 2023) to obtain an initial set of genes associated with glyphosate exposure. To enhance the specificity and biological relevance of the dataset, only genes with ≥4 curated chemical-gene interactions were retained, thereby prioritizing consistently reported associations and reducing the inclusion of low-evidence signals. The resulting gene list was exported in tabular format for downstream analyses.

A total of 81 genes met the predefined selection criterion. Subsequently, gene identity and species annotation were verified against UniProt to ensure inclusion of validated human genes. Three entries (*PAX2A, TGFB1A*, and *BAXA*) were excluded due to lack of confirmation as human genes. The final curated dataset comprised 78 genes, which were used for subsequent integrative and network-based analyses.

This filtering strategy was designed to balance sensitivity and specificity, prioritizing reproducible gene–chemical associations.

#### 2.2.2 GeneShot plataform

Gene prioritization based on literature-derived associations was performed using the GeneShot platform (Lachmann et al., 2019), developed by the Ma’ayan Laboratory at the Icahn School of Medicine at Mount Sinai. This tool identifies gene-term associations through co-occurrence analysis between biomedical concepts and gene names within PubMed-indexed scientific literature, enabling the capture of genes recurrently reported in the context of glyphosate exposure.

All retrieved genes were initially included to preserve sensitivity to literature-derived signals, resulting in a total of 92 genes, which were exported in tabular format for downstream processing. Subsequently, gene identity and species annotation were verified to ensure consistency with *Homo sapiens*. One gene (*TRP53*) was excluded due to lack of correspondence with human gene annotation. The final dataset comprised 91 genes.

This approach allowed the incorporation of literature-driven evidence, complementing curated databases by capturing broader associative signals, while subsequent filtering ensured taxonomic consistency and data reliability.

#### 2.2.3 GeneCards database

In the GeneCards database (Stelzer et al., 2016), human genes associated with the search term “glyphosate” were identified. To prioritize genes with higher levels of functional characterization and support across multiple data sources, a selection criterion based on a GeneCards Inferred Functionality Score (GIFtS) > 1.0 was applied. This threshold enabled the retention of genes with a minimum level of integrated functional evidence, reducing the inclusion of poorly annotated or weakly supported entries.

Based on this criterion, 20 genes were selected for inclusion in the analysis. Gene identity and species annotation were subsequently verified to ensure consistency with *Homo sapiens*.

One gene (*PEPC*) was excluded because it did not correspond to a human annotation, leaving a total of 19 genes in the curated set. This procedure allowed for the incorporation of integrated functional information, complementing both the curated evidence derived from the literature, and contributing to the construction of a more robust gene set for subsequent analyses.

#### 2.2.4 ChatGPT 4.0

An AI-assisted literature retrieval and prioritization approach was employed to complement curated database searches and identify additional human genes reported in association with glyphosate exposure. While curated resources such as CTD, GeneShot, and GeneCards offer high reliability, their coverage depends on prior formal curation or functional annotation, and may therefore not capture genes supported by more recent or dispersed literature; the AI-assisted step was included as an exploratory search layer to address this gap, without replacing the curated databases. Retrieval was performed using the ChatGPT web interface (OpenAI), GPT-4 model, in April 2025, through two sequential instructions: the first requested genes reported in association with glyphosate exposure in humans or in experimental models with translational relevance to human biology; the second requested that the identified genes be organized using a composite scoring scheme (1–5 scale) based on frequency of occurrence in the literature, consistency of reported associations, reproducibility across studies, and magnitude of the observed effects. As outputs generated through a conversational AI interface are not strictly deterministic, methodological transparency in this step relies on reporting the model version, interface, date of use, and the substantive content of the instructions provided, rather than on exact output reproducibility. To minimize potential biases associated with AI-assisted tools, the resulting gene list underwent manual curation and cross-validation against three independently curated databases: CTD, GeneShot, and GeneCards. A gene was retained in the integrated core gene set if it was identified in at least two of the four independent sources considered (CTD, GeneShot, GeneCards, and AI-assisted retrieval). A total of 34 genes directly or indirectly associated with glyphosate were initially identified through ChatGPT; after removal of one duplicated gene (SOD), a final set of 33 genes was obtained, of which 16 (48.5%) were independently corroborated by at least one of the three curated databases and were retained in the integrated gene set, while the remaining 17 genes, supported exclusively by AI-assisted retrieval, were excluded. The use of AI-assisted tools was restricted exclusively to information retrieval and prioritization and did not replace conventional bioinformatic or statistical analyses.

#### 2.2.5 Integration of sources and analysis of gene intersections

Overlaps among the independent gene lists derived from CTD, GeneShot, GeneCards, and ChatGPT were identified using Venny 2.1 (CNB-CSIC; https://bioinfogp.cnb.csic.es/tools/venny/, accessed 8 September 2025). Prior to integration, all gene identifiers (Entrez, UniProt, and Ensembl IDs) were standardized to official HGNC symbols for *Homo sapiens* (Braschi et al., 2019), updating obsolete or ambiguous nomenclature and removing duplicates to ensure consistency across sources. The resulting Venn diagram was exported in PNG format for documentation. The core gene set was defined as genes present in at least two of the four sources (≥2), based on the premise that convergence across independent datasets increases the robustness and reliability of gene-glyphosate associations while reducing source-specific false positives and prioritizing reproducible associations.

#### 2.2.6 Identification of differentially expressed genes from GEO datasets using GEO2R

To complement evidence from curated databases and literature mining, publicly available transcriptomic datasets were searched in the Gene Expression Omnibus (GEO) (Clough and Barrett, 2016; Edgar et al., 2002) using “glyphosate” as the search term. Only datasets including glyphosate-exposed samples with corresponding non-exposed controls, and compatible with GEO2R (NCBI), were considered. Two independent studies met these criteria: human MCF-7 breast cancer cells exposed, among other compounds, to glyphosate (Platform GPL16791, Series GSE87701) (Mesnage et al., 2017b), and a human pluripotent stem cell-derived neural tissue model incorporating vascular networks and microglia, designed to evaluate neurotoxic responses to different compounds, including glyphosate (Platform GPL16791, Series GSE63935) (Schwartz et al., 2015). Differential expression analysis was performed using GEO2R, comparing glyphosate-treated samples with their respective untreated controls; for the study by Schwartz et al. (2015) Only the day-21 comparison was considered, contrasting exposed samples with time-matched day-21 controls to reduce temporal heterogeneity and improve biological comparability.

DEGs were selected based on the adjusted P-value after multiple-testing correction (Benjamini-Hochberg FDR <0.05) (Benjami et al., 1995); no additional log2 fold-change threshold was applied, and log2 fold-change values were retained for descriptive purposes only. No overlap was observed between transcriptome-derived genes and those retrieved from the other sources, yielding eight unique DEGs (three from the MCF-7 model and five from the neural tissue model). Given its nature as pseudogene, one of these DEGs, *FER1L4*, was not directly usable in the protein-protein interaction analysis; instead, its three documented protein-coding target genes (*PTEN, E2F1*, and *DNMT3B*) were incorporated as proxies of its potential regulatory effects, resulting in a final contribution of ten genes from the transcriptomic analysis.

This transcriptomic evidence added an independent experimental dimension to the integrative model, complementing the core gene set built from the convergence of curated databases, literature mining, and AI-assisted tools, and supporting the subsequent network construction, functional enrichment, and topological prioritization analyses.

This multilayered approach yielded a biologically coherent gene set, consistent with contemporary network biology and multi-omics strategies applied to complex diseases and environmental exposures (Barabási et al., 2011; Hasin et al., 2017), moving beyond a purely descriptive, gene-centered approach toward a more integrative interpretation of the molecular mechanisms potentially associated with glyphosate exposure.

Complete gene lists from each independent source, along with their selection criteria, are provided in Supplementary Table S1; DEGs identified through GEO2R are presented in Supplementary Table S2.

### 2.3 Integrative network and functional analysis

#### 2.3.1 Protein–protein interaction network analysis (PPI)

For construction of the protein–protein interaction (PPI) network, the previously consolidated core gene set obtained through multi-source integration was used and complemented with eight additional genes derived from GEO2R transcriptomic analyses identified in independent studies (Mesnage et al., 2017b; Schwartz et al., 2015).

The PPI network was generated using STRING version 12.0 (Search Tool for the Retrieval of Interacting Genes/Proteins) (Szklarczyk et al., 2019) using *Homo sapiens* as the reference organism and a medium-confidence interaction score of 0.400. The resulting network integrated physical and functional relationships among genes associated with glyphosate exposure. To address potential bias related to network density and the prioritization of generic high-degree genes, hub-gene identification was interpreted in conjunction with independent analytical layers, including functional enrichment, gene-disease and phenotype associations, and upstream transcriptional regulator analysis.

The network was subsequently imported into Cytoscape version 3.6.1 (Shannon et al., 2003), where visualization, topological analysis, and graphical refinement were performed.

Because STRING is primarily restricted to protein-protein interactions, the pseudogene *FER1L4* is not directly represented in this platform. To incorporate this element, coding target genes supported by experimental or predictive evidence were compiled from the scientific literature (Li et al., 2022; Xia et al., 2015; Xia et al., 2019). A PPI subnetwork was generated from these targets in STRING, and *FER1L4* was subsequently added in Cytoscape as a regulatory node connected to its targets through non-PPI edges, including ceRNA relationships, co-expression links, or RNA-binding protein (RBP) interactions.

#### 2.3.2 Hub gene prioritization

Key gene identification within the network was performed using the cytoHubba plugin (Chin et al., 2014), applying five topological algorithms: MCC (Maximal Clique Centrality), Degree, Betweenness Centrality, DMNC (Density of Maximum Neighborhood Component), and Closeness. For each algorithm, the top ten ranked genes were selected, enabling the identification of highly connected and structurally relevant nodes within the network.

Hub genes were prioritized using a consensus score that integrated recurrence across algorithms and mean rank position, giving greater weight to genes consistently identified across multiple centrality metrics. To visualize connectivity hierarchy, a red-to-yellow color gradient was applied (red = higher centrality; yellow = lower centrality), following previously described approaches (Christodoulou and Papanicolaou, 2023). Hub analysis was restricted to the PPI subnetwork composed of protein-coding genes, excluding non-protein interactions associated with *FER1L4,* to maintain methodological consistency in the topological analysis.

#### 2.3.3 Functional enrichment analysis

Functional enrichment analysis leveraged the “Analyze” function within the STRING platform, interrogating multiple annotation levels: Gene Ontology (GO) (Aleksander et al., 2023) categories: *Biological Process* (BP) and *Molecular Function* (MF), alongside pathway-level annotations from the Kyoto Encyclopedia of Genes and Genomes (KEGG) (Kanehisa and Goto, 2000). Beyond functional categories, the analysis extended to disease and phenotypic associations through STRING-integrated resources, including Disease Associations and the Human Phenotype Ontology (HPO) (Köhler et al., 2021).

**FIGURE 1:**
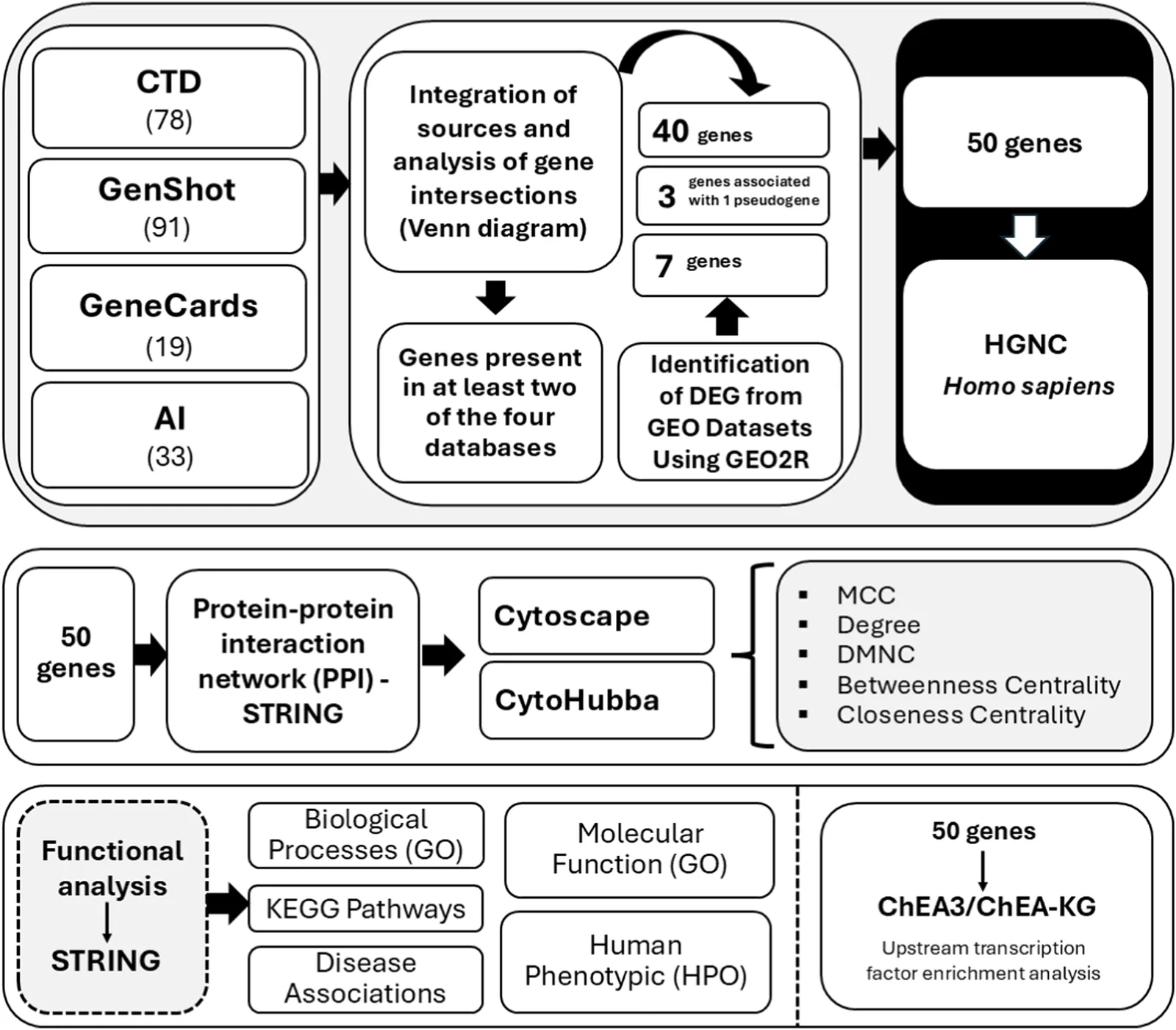
General workflow of data curation and analytical integration. Schematic representation of the methodological framework used for the identification and integration of genes associated with glyphosate exposure. The workflow includes querying multiple bioinformatics databases, artificial intelligence–assisted information retrieval, standardization of gene identifiers according to HGNC, and integration through overlap analysis, complemented by transcriptomic evidence. Subsequently, selected genes were subjected to protein–protein interaction network analysis, functional enrichment, and upstream transcriptional regulatory analysis to construct an integrative framework for mechanistic interpretation of glyphosate-associated biological effects.

Significantly enriched terms were identified based on a false discovery rate (FDR)-adjusted *p*-value to account for multiple testing and control type I error (Benjami et al., 1995). The results were subsequently visualized using bubble charts, which summarize the most significantly enriched terms across each functional category, facilitating the interpretation of the biological processes, molecular functions, and pathways potentially affected by glyphosate exposure (Figure 1).

#### 2.3.4 Analysis of transcription regulators (UPSTREAM)

Upstream transcription factor enrichment analysis was performed using ChEA3 (https://maayanlab.cloud/chea3/) (Keenan et al., 2019), inputting the 50-gene core set *via* HGNC approved gene symbols and prioritizing candidates according to the Integrated MeanRank (Byrd et al., 2025). As ChEA3 is a continuously updated, web-based resource that does not allow version control or archival access to prior database states, transcription factor prioritization may vary over time as new regulatory evidence is incorporated. Rather than treating this as a limitation to be avoided, we used it as an opportunity to empirically test the temporal stability of the prioritization: the analysis was performed independently at two time points 3 weeks apart (April 30 and 18 May 2026), using the identical 50 gene input set. At each time point, the top 20 transcription factors were retained based on their Integrated MeanRank; those consistently identified at both time points were considered temporally stable candidates for subsequent interpretation, whereas those detected at only one time point were excluded. Among the temporally stable candidates, their average Integrated MeanRank across both analyses was used to establish the final prioritization. Building on this ranking, each transcription factor underwent biological contextualization through a targeted literature review examining its reported functional roles.

## 3 Results

### 3.1 Gene set integration and PPI network construction

**FIGURE 2:**
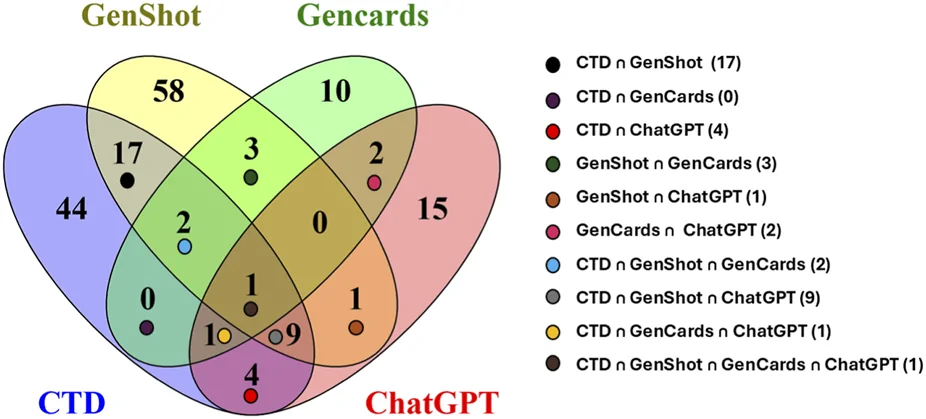
Overlap of genes associated with glyphosate across multiple data sources. Venn diagram illustrating the overlap of genes associated with glyphosate exposure retrieved from GeneCards, the Comparative Toxicogenomics Database (CTD), GeneShot, and artificial intelligence–assisted curation (ChatGPT). Genes present in at least two independent sources were selected to define the core gene set used for subsequent analyses. This integrative approach enhances the robustness of gene selection by prioritizing consistently reported associations across heterogeneous datasets.

From an initial pool of 221 candidate genes retrieved from four independent sources associated with glyphosate exposure (CTD, GeneShot, GeneCards, and AI-assisted retrieval), the integration process resulted in the identification of a core set of 50 genes, defined by their presence in at least two independent sources (Figure 2; Table 1).

**TABLE 1:** Core set of glyphosate-associated genes selected for integrative network analysis.

Gene	Definition	Affected function, organ, or system
CCND1	G1/S-specific cyclin-D1	Cell cycle, apoptosis, or cell death
TP53	Cellular tumor antigen p53	
BAX	Apoptosis regulator BAX, Bcl-2-like protein 4, Bcl2-L-4	
BCL2	Apoptosis regulator Bcl-2	
CASP3	Caspase 3	
CASP9	Caspase 9	
CDKN1A	Cyclin-dependent kinase inhibitor 1	
E2F1	E2F transcription factor 1	
DNMT1	DNA (cytosine-5)-methyltransferase 1	Epigenetic regulation
DNMT3A	DNA (cytosine-5)-methyltransferase 3A	
TET3	Methylcytosine dioxygenase TET3	
DNMT3B	DNA (cytosine-5)-methyltransferase 3B	
CAT	Catalase	Oxidative stress - stress response - inflammation
SOD 1	Superoxide Dismutase 1	
SOD 2	Superoxide Dismutase 2	
IL 6	Interleukin 6	
IL 10	Interleukin 10	
TNF	Tumor Necrosis factor	
EIF2S1	Eukaryotic translation initiation factor 2 Subunit 1	
HMOX1	Heme Oxygenase 1	
IL1B	Interleukin 1 Beta	
NFE2L2	Nuclear factor erythroid 2-related factor 2	
AR	Androgen receptor	Hormone signaling- endocrine disruption
ESR 1	Estrogen receptor 1	
ESR 2	Estrogen receptor 2	
PGR	Progesterone receptor	
STAR	Steroidogenic Acute regulatory protein mitochondrial	
CYP19A1	Aromatase - cytochrome P450 19A1, estrogen synthase	
GREB1	Protein GREB1, gene regulated in breast cancer 1 protein	
HOXA10	Homeobox protein Hox-A10	
ABCB1	ATP binding cassette subfamily B Member 1	Xenobiotic metabolism - transport
ABCG2	ATP binding cassette subfamily G Member 2	
GLUL	Glutamine synthetase - Glutamate--ammonia ligase	
CYP3A4	Cytochrome P450 family 3 Subf A Member 4	
GPT	Alanine aminotransferase 1. Glutamate pyruvate transaminase 1	
PPOX	Protoporphyrinogen Oxidase	
FOS	Transcription factor AP-1 subunit c-Fos	
MAPK1	Mitogen-activated protein kinase 1	
HSP90AA1	Heat Shock protein 90 alpha family class A Member 1	
WNT5A	Protein Wnt-5A	
PTEN	Phosphatase and tensin homolog	
ESM1	Endothelial cell specific molecule 1	Vascular - endothelial integrity - tissue remodeling
FLT1	Fms-related receptor tyrosine kinase 1	
TJP1	Tight Junction protein 1	
APOD	Apolipoprotein D	
ALB	Albumin	
CD93	CD93 molecule	
PLVAP	Plasmalemma vesicle associated protein	
EMILIN1	Elastin microfibril interfacer 1	
ACHE	Acetylcholinesterase	Other processes

Complete list of the core set of genes associated with glyphosate exposure selected for downstream analyses. Genes were prioritized through overlap across multiple independent sources, including CTD, GeneCards, GeneShot, and artificial intelligence - assisted curation, and were complemented by differentially expressed genes identified from GEO2R transcriptomic datasets. Gene identifiers were standardized according to HGNC, nomenclature. This final gene set was used for subsequent network construction, functional enrichment, and regulatory analyses.

Of these, 40 genes were derived from the convergence of bioinformatics platforms (Supplementary Table S1), while ten additional genes were incorporated through independent transcriptomic evidence (GEO2R): *ESM1, FLT1, CD93, PLVAP*, and *EMILIN1* from a neural tissue model, and *GREB1, FER1L4*, and *APOD* from a human breast cancer cell model (Mesnage et al., 2017b; Schwartz et al., 2015) (Supplementary Table S2). Given its nature as a pseudogene, *FER1L4* did not enter the protein-protein interaction (PPI) analysis directly; instead, its three documented protein-coding target genes: *PTEN, E2F1*, and *DNMT3B*, served as proxies to capture its potential indirect regulatory effects, accounting for the ten genes ultimately incorporated from the transcriptomic analysis (Table 2).

**TABLE 2:** Experimentally supported ceRNA regulatory interactions of the lncRNA *FER1L4*in humans.

Intermediate miRNA	Encoding target/protein	Function	Pathology	Ref.
miR-106a-5p	*PTEN*	Tumor suppressor gene	Gastric cancer	Xia et al. (2015)
miR-372	*E2F1*	Nuclear transcription factor	High-grade gliomas	Xia et al. (2019)
Not recorded	*DNMT3B*	Has been described as a tumor suppressor in cancer	Colorectal cancer (CRC)	Li et al. (2022)

Summary of experimentally reported competing endogenous RNA (ceRNA) interactions involving the lncRNA *FER1L4*in human studies. The table lists target genes modulated by *FER1L4*and their corresponding references, highlighting its potential role in post-transcriptional regulation through miRNA-mediated mechanisms.

**FIGURE 3:**
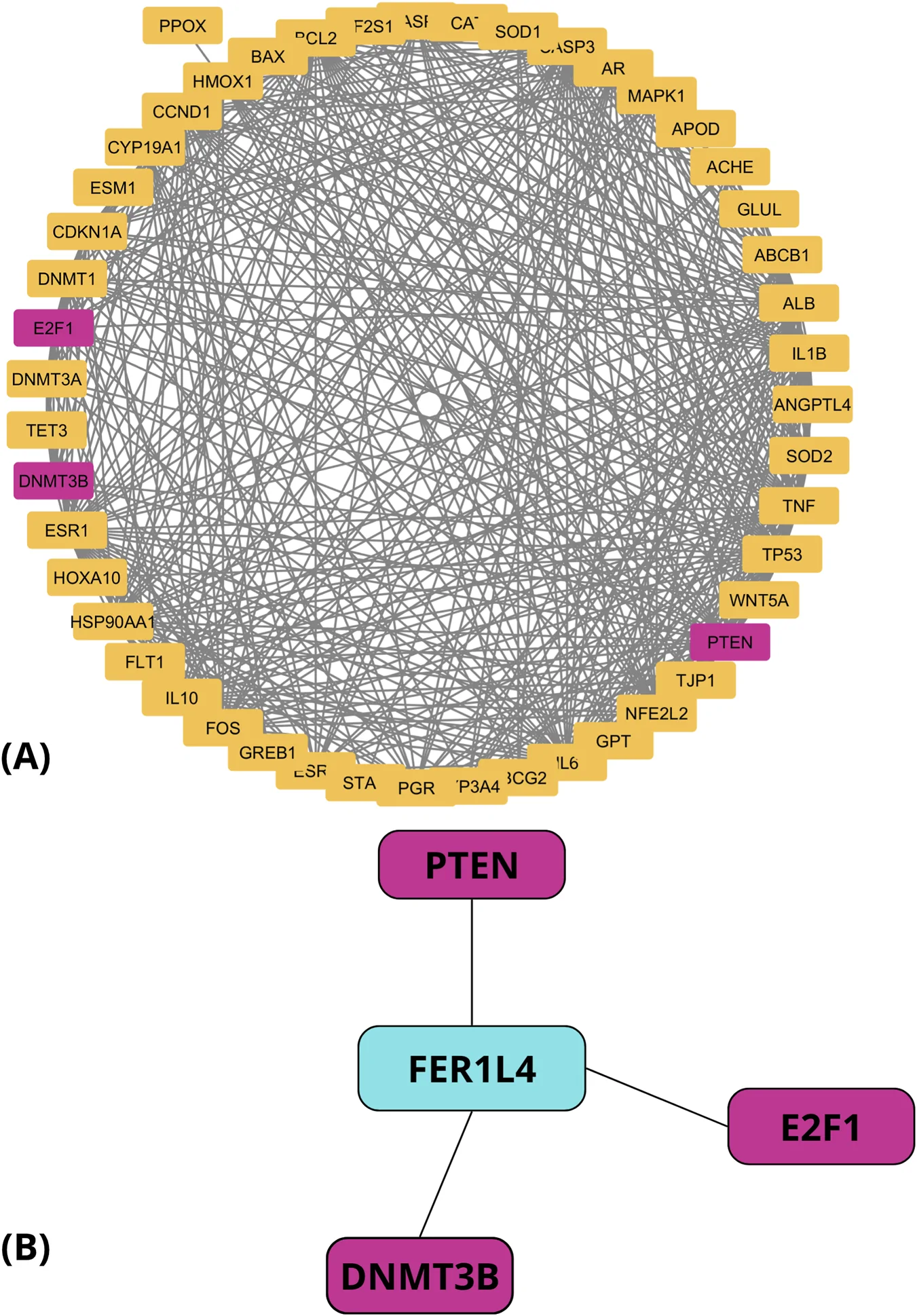
Protein–protein interaction network and regulatory integration of glyphosate-associated genes. Protein–protein interaction (PPI) network constructed for glyphosate-associated genes. **(A)** Network visualization in Cytoscape, highlighting the topological organization and connectivity patterns of the gene network. **(B)** Subnetwork showing protein-coding genes associated with the pseudogene *FER1L4*, incorporated to represent potential regulatory interactions beyond protein–protein associations. These representations illustrate the interconnected structure of the gene network and its integration with non-coding regulatory elements, supporting a multi-layered view of glyphosate-associated molecular responses.

The resulting PPI network comprised 50 nodes and 446 edges (average node degree: 17.8; average local clustering coefficient: 0.775), forming a densely connected structure (Figure 3). This connectivity significantly exceeded random expectations, as only 177 edges would be expected for a gene set of identical size (PPI enrichment p-value <1.0 × 10^−16^), supporting a high degree of functional interrelatedness among the analyzed genes.

**FIGURE 4:**
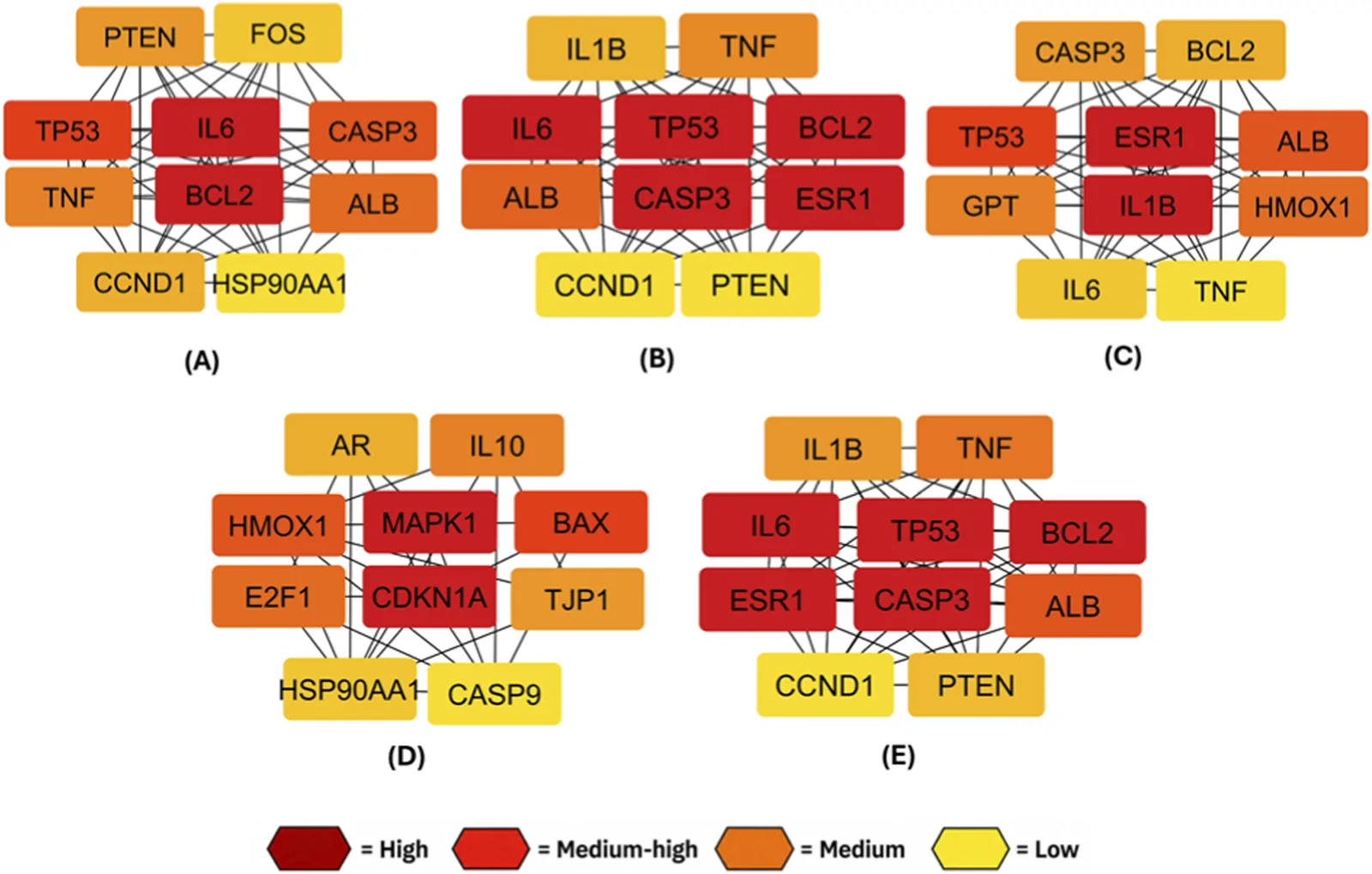
Identification of key hub genes within the glyphosate-associated protein–protein interaction network. Identification of the top 10 hub genes based on network centrality analysis using five topological algorithms implemented in the cytoHubba plugin of Cytoscape: **(A)** MCC (Maximal Clique Centrality), **(B)** Degree, **(C)** Betweenness Centrality, **(D)** DMNC (Density of Maximum Neighborhood Component), and **(E)** Closeness Centrality.

Centrality analysis identified key nodes within the network. The MCC algorithm highlighted *IL6, BCL2*, and *TP53* as the most central genes. Similarly, the Degree algorithm ranked *TP53*, *CASP3*, *BCL2, IL6 and ESR1,* among the top nodes, while Betweenness, DMNC, and Closeness identified *ESR1, IL1B,* and *TP53*; *MAPK1, CDKN1A*, and *BAX*; and *TP53*, *CASP3, BCL2, IL6 and ESR1,* respectively, as highly relevant genes (Table 3; Figure 4).

**TABLE 3:** Consensus hub genes identified across multiple topological algorithms in the *cytoHubba* analysis.

Rank	MCC	Degree	Betweenness	DMNC	Closeness
1	*IL6*	*TP53*	*ESR1*	*MAPK1*	*TP53*
2	*BCL2*	*CASP3*	*IL1B*	*CDKN1A*	*CASP3*
3	*TP53*	*BCL2*	*TP53*	*BAX*	*BCL2*
4	*CASP3*	*IL6*	*ALB*	*HMOX1*	*IL6*
5	*ALB*	*ESR1*	*HMOX1*	*E2F1*	*ESR1*
6	*TNF*	*ALB*	*GPT*	*IL10*	*ALB*
7	*PTEN*	*TNF*	*CASP3*	*TJP1*	*TNF*
8	*CCND1*	*IL1B*	*BCL2*	*AR*	*IL1B*
9	*FOS*	*CCND1*	*IL6*	*HSP90AA1*	*PTEN*
10	*HSP90AA1*	*PTEN*	*TNF*	*CASP9*	*CCND1*

Top-ranked hub genes identified using five topological algorithms implemented in the cytoHubba plugin of Cytoscape. Genes consistently ranked by at least four algorithms were defined as consensus hub genes, reflecting robust centrality within the protein–protein interaction network. Abbreviations: MCC, maximal clique centrality; DMNC, density of maximum neighborhood component.

**FIGURE 5:**
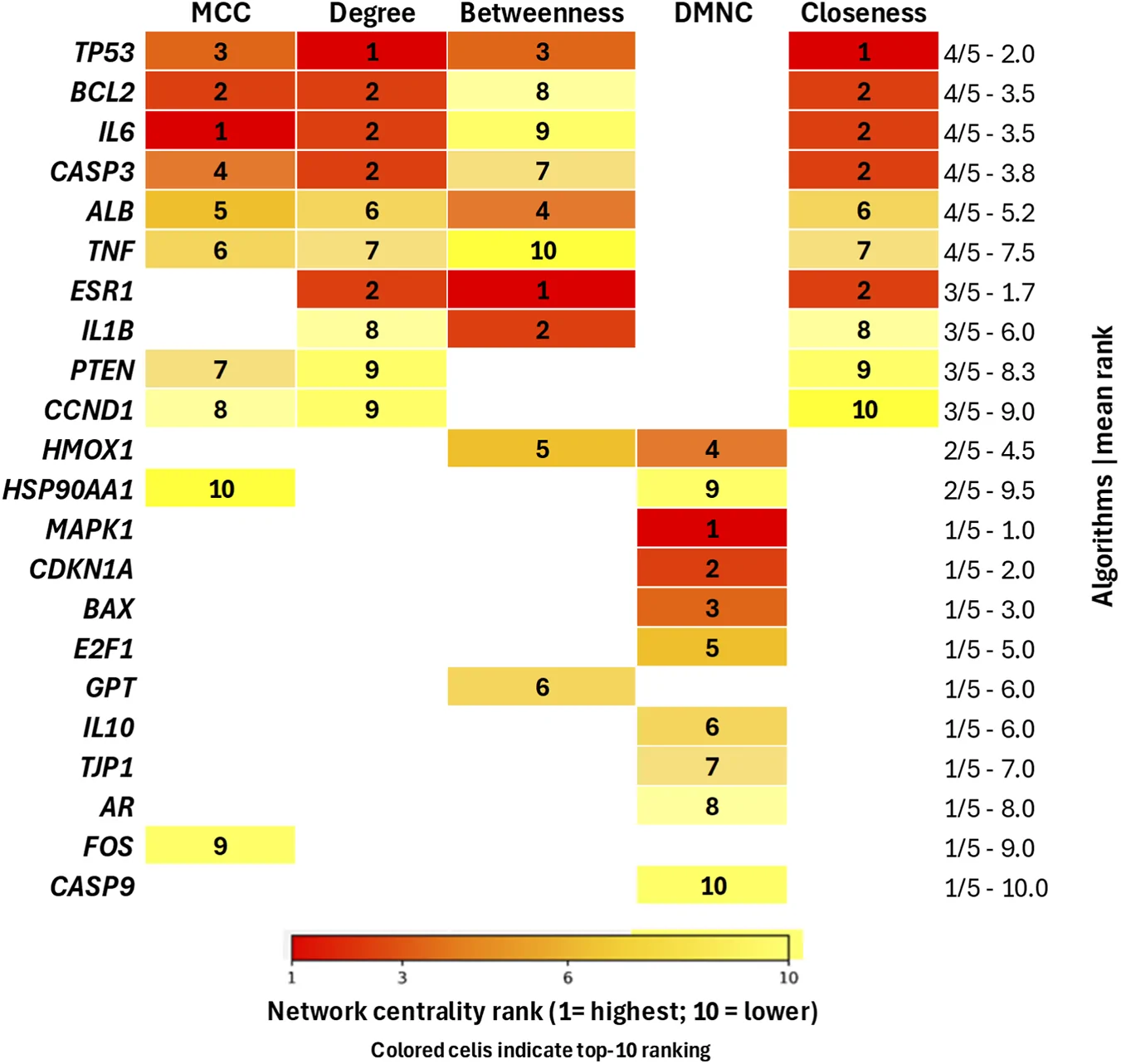
Consensus identification and connectivity hierarchy of prioritized hub genes. Integrated analysis of hub gene prioritization across multiple centrality algorithms. Heatmap representing the connectivity hierarchy of genes across algorithms, where each row corresponds to a gene and each column to a topological method. Colors indicate relative connectivity rank: red (high), orange (medium-high), bright yellow (medium), and light yellow (low).

Integration of the results obtained from the different topological algorithms identified six genes recurrently ranked in at least four of them (*TP53, BCL2, IL6, CASP3, ALB*, and *TNF*), which were prioritized as critical nodes (hub genes) due to their high connectivity and central position within the network (Figure 5). These findings indicate a hierarchical network organization characterized by a reduced subset of highly interconnected genes occupying central positions across multiple topological metrics.

### 3.2 Functional enrichment analysis

**FIGURE 6:**
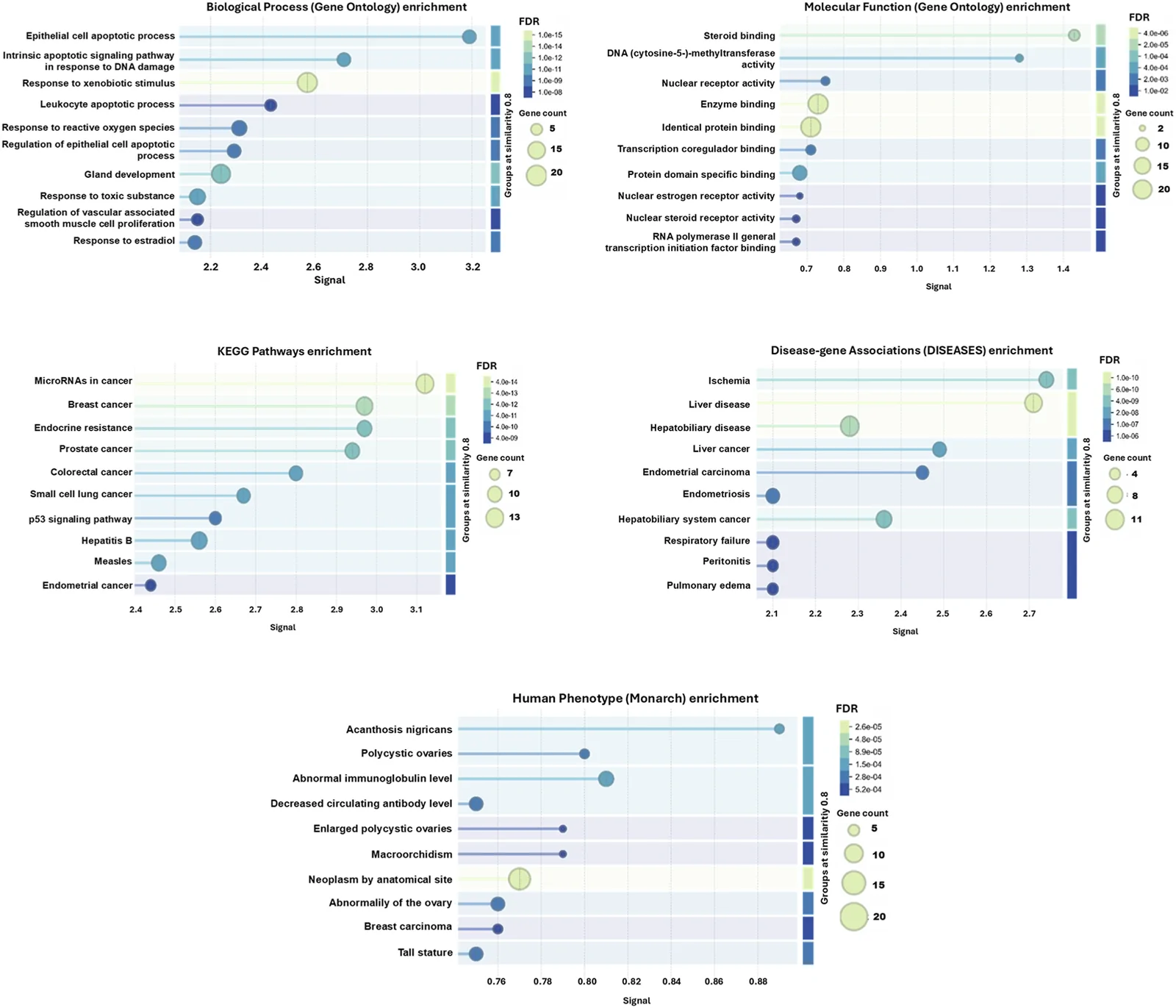
Integrated functional enrichment analysis across biological processes, molecular functions, pathways, and disease associations. Integrated bubble plot summarizing functional enrichment results across multiple annotation levels, including Gene Ontology biological processes (GO: BP), molecular functions (GO:MF), KEGG pathways, gene–disease associations, and Human Phenotype Ontology (HPO). Each panel displays the top enriched terms within each category. The x-axis represents enrichment signal strength, while the y-axis lists the significantly enriched terms. Bubble size corresponds to the number of genes associated with each term, and color intensity reflects statistical significance based on false discovery rate (FDR), with lighter colors indicating lower FDR values. This integrative representation highlights the convergence of enriched terms related to cellular stress response, apoptosis, endocrine signaling, cancer-associated pathways, and disease phenotypes, supporting a multi-level functional interpretation of the gene set.

Functional enrichment analysis of Gene Ontology terms related to biological processes revealed a significant overrepresentation of pathways associated with cellular responses to chemical stress, apoptosis, endocrine signaling, and tissue regulation (Figure 6; Table 4). Among the most significantly enriched terms were response to xenobiotic stimulus (FDR = 1.46 × 10^−15^) and *response to toxic substance* (FDR = 2.99 × 10^−10^), supporting the activation of cellular defense mechanisms against potentially harmful exogenous compounds. Consistently, *response to reactive oxygen species* (FDR = 3.17 × 10^−10^) indicated the involvement of pathways linked to oxidative stress.

**TABLE 4:** STRING-based functional enrichment of glyphosate-associated genes across biological annotation levels.

Categories	Description	Count in network	%	Strength	Signal	False discovery rate
4.1. Biological process	*Epithelial cell apoptotic process*	9/52	17.3	1.83	3.19	2.11e-11
	*Intrinsic apoptotic signaling pathway in response to DNA damage*	9/75	12.0	1.67	2.71	2.94e-10
	*Response to xenobiotic stimulus*	19/422	4.50	1.25	2.57	1.46e-15
	*Leukocyte apoptotic process*	7/45	15.6	1.79	2.43	1.22e-08
	*Response to reactive oxygen species*	11/169	6.51	1.41	2.31	3.17e-10
	*Regulation of epithelial cell apoptotic process*	9/106	8.49	1.52	2.29	3.40e-09
	*Gland development*	17/419	4.06	1.20	2.24	3.40e-13
	*Response to toxic substance*	12/229	5.24	1.31	2.15	2.99e-10
	*Regulation of muscle cell proliferation*	7/61	11.5	1.66	2.15	6.13e-08
	*Response to estradiol*	9/121	7.44	1.47	2.14	8.44e-09
4.2. Molecular function	*Steroid binding*	7/101	6.93	1.44	1.43	1.22e-05
	*DNA (cytosine-5)-methyltransferase activity*	3/3	100	2.60	1.28	2.50e-04
	*Nuclear receptor activity*	4/53	7.55	1.47	0.75	4.90e-03
	*Enzyme binding*	22/2084	1.06	0.62	0.73	4.16e-06
	*Identical protein binding*	22/2144	1.03	0.61	0.71	4.72e-06
	*Transcription coregulator binding*	5/114	4.39	1.24	0.71	4.90e-03
	*Protein domain specific binding*	11/695	1.58	0.79	0.68	8.30e-04
	*Nuclear estrogen receptor activity*	2/3	66.7	2.42	0.68	1.19e-02
	*Nuclear steroid receptor activity*	3/25	12.0	1.67	0.67	1.07e-02
	*RNA polymerase II general transcription initiation factor binding*	3/25	12.0	1.67	0.67	1.07e-02
4.3. KEGG pathways	*microRNAs in cancer*	13/159	8.18	1.51	3.12	4.72e-14
	*Breast cancer*	12/146	8.22	1.51	2.97	4.71e-13
	*Endocrine resistance*	10/94	10.6	1.62	2.97	7.63e-12
	*Prostate cancer*	10/97	10.3	1.61	2.94	8.17e-12
	*Colorectal cancer*	9/82	11.0	1.64	2.80	6.43e-11
	*Small cell lung cancer*	9/92	9.78	1.59	2.67	1.31e-10
	*p53 signaling pathway*	8/72	11.1	1.64	2.60	6.12e-10
	*Hepatitis B*	11/158	6.96	1.44	2.56	2.03e-11
	*Measles*	10/137	7.30	1.46	2.46	1.28e-10
	*Endometrial cancer*	7/58	12.1	1.68	2.44	4.50e-09
4.4 disease-gene associations	*Ischemia*	7/30	23.3	1.96	2.74	2.75e-09
	*Liver disease*	10/97	10.3	1.61	2.71	1.12e-10
	*Liver cancer*	6/21	28.6	2.05	2.49	2.80e-08
	*Endometrial carcinoma*	5/9	55.6	2.34	2.45	7.66e-08
	*Hepatobiliary system cancer*	8/68	11.8	1.67	2.36	8.92e-09
	*Hepatobiliary disease*	11/168	6.55	1.41	2.28	5.24e-10
	*Endometriosis*	6/34	17.6	1.84	2.10	2.66e-07
	*Respiratory failure*	5/17	29.41	2.06	2.10	5.22e-07
	*Peritonitis*	4/5	80.0	2.50	2.10	9.93e-07
	*Pulmonary edema*	4/5	80.0	2.50	2.10	9.93e-07
4.5. Human phenotype	*Acanthosis nigricans*	4/38	10.5	1.62	0.89	2.1e-03
	*Abnormal immunoglobulin level*	7/211	3.32	1.12	0.81	1.3e-03
	*Polycystic ovaries*	4/48	8.33	1.52	0.80	3.6e-03
	*Enlarged polycystic ovaries*	3/17	17.6	1.84	0.79	5.2e-03
	*Macroorchidism*	3/17	17.6	1.84	0.79	5.2e-03
	*Neoplasm by anatomical site*	13/732	1.78	0.84	0.77	2.7e-04
	*Abnormality of the ovary*	6/165	3.64	1.16	0.76	2.3e-03
	*Breast carcinoma*	4/54	7.41	1.47	0.76	4.7e-03
	*Decreased circulating antibody level*	6/169	3.55	1.15	0.75	2.5e-03
	*Tall stature*	6/170	3.53	1.14	0.75	2.5e-03

Summary of the top 10 enriched terms identified through STRING-based functional enrichment analysis across multiple annotation categories, including Gene Ontology biological processes (GO:BP); molecular functions (GO:MF); KEGG pathways; gene-disease associations, and Human Phenotype Ontology (HPO).

Terms are ranked according to statistical significance based on false discovery rate (FDR < 0.05). The table includes the number of genes associated with each term, enrichment strength, and signal score as provided by STRING. This integrated analysis highlights the convergence of functional categories related to cellular stress response, apoptosis, endocrine signaling, cancer-associated pathways, and disease phenotypes.

Apoptosis-related processes were also prominently enriched, including *epithelial cell apoptotic process* (FDR = 2.11 × 10^−11^), *intrinsic apoptotic signaling pathway in response to DNA damage* (FDR = 2.94 × 10^−10^), *regulation of epithelial cell apoptotic process* (FDR = 3.40 × 10^−9^), and *leukocyte apoptotic process* (FDR = 1.22 × 10^−8^). Collectively, these findings suggest a central role for programmed cell death and its regulatory mechanisms within the analyzed gene set. Terms related to hormonal signaling and tissue organization were also identified, including *response to estradiol* (FDR = 8.44 × 10^−9^) and *gland development* (FDR = 3.40 × 10^−13^), supporting the involvement of endocrine pathways and cellular differentiation processes.

Finally, *regulation of muscle cell proliferation* (FDR = 6.13 × 10^−8^) suggested an additional contribution of pathways related to cellular proliferation and tissue remodeling. Overall, the observed functional profile points to a convergence between chemical stress response, apoptosis, hormonal signaling, and adaptive cellular regulation.

Functional enrichment analysis of Gene Ontology terms related to molecular function revealed a significant overrepresentation of activities associated with hormonal signaling, epigenetic regulation, transcriptional control, and protein–protein interactions (Table 4; Figure 6). Among the most significantly enriched terms were *steroid binding* (FDR = 1.22 × 10^−5^) and *DNA (cytosine-5)-methyltransferase activity* (FDR = 2.50 × 10^−4^), supporting the involvement of mechanisms linked to endocrine signaling and epigenetic modification. Notably, *DNA (cytosine-5)-methyltransferase activity* showed the highest enrichment strength (2.60) and complete representation within the network (3 of 3 genes), suggesting a highly specific functional signal. Functions related to nuclear receptors were also identified, including *nuclear receptor activity* (FDR = 4.90 × 10^−3^), *nuclear estrogen receptor activity* (FDR = 0.0119), and *nuclear steroid receptor activity* (FDR = 0.0107), supporting the involvement of hormone-dependent gene regulatory pathways.

Additional terms associated with binding and protein interactions included *enzyme binding* (FDR = 4.16 × 10^−6^), *protein domain specific binding* (FDR = 8.30 × 10^−4^), and identical protein binding (FDR = 4.72 × 10^−6^), reflecting the central role of molecular interactions within the network. Among these, enzyme binding showed the largest number of associated genes (22 of 2084), indicating broad functional participation. Finally, terms related to transcriptional regulation. such as *transcription coregulator binding* (FDR = 4.90 × 10^−3^) and *RNA polymerase II general transcription initiation factor binding* (FDR = 0.0107), further support the involvement of transcriptional control mechanisms within the analyzed gene set.

KEGG pathway enrichment analysis revealed a significant overrepresentation of routes mainly associated with cancer, endocrine regulation, and cellular stress responses (Table 4; Figure 6). The most significantly enriched pathways included *microRNAs in cancer* (FDR = 4.72 × 10^−14^), *breast cancer* (FDR = 4.71 × 10^−13^), and *endocrine resistance* (FDR = 7.63 × 10^−12^), alongside multiple neoplastic pathways such as prostate cancer, colorectal cancer, small cell lung cancer, and endometrial cancer, collectively indicating a consistent concentration of gene networks involved in tumorigenesis and hormone-related signaling.

The *p53 signaling pathway* (FDR = 6.12 × 10^−10^) was also significantly enriched, supporting the involvement of mechanisms related to DNA damage response, cell-cycle control. and apoptosis. Additional pathways associated with infectious diseases, such as *Hepatitis B* (FDR = 2.03 × 10^−11^), and *Measles* (FDR = 1.28 × 10^−10^), included, likely reflecting shared molecular processes involving immune response, inflammation, and cellular stress signaling.

In terms of enrichment magnitude and gene representation, *endometrial cancer* (12.07%. strength = 1.68), *p53 signaling pathway* (11.11%. strength = 1.64), and *colorectal cancer* (10.98%, strength = 1.64) showed the highest proportions. Likewise, *microRNAs in cancer* (13 genes), and *breast cancer* (12 genes) contained the largest numbers of associated genes within the network. These findings suggest a convergence between proliferative dysregulation, endocrine alterations, and cellular stress-response mechanisms.

Gene-disease association analysis revealed significant enrichment of conditions mainly related to hepatobiliary disorders, neoplastic processes, and systemic pathologies (Table 4; Figure 6). Among the most significantly associated diseases were *liver disease* (FDR = 1.12 × 10^−10^), *hepatobiliary disease* (FDR = 5.24 × 10^−10^), *hepatobiliary system cancer* (FDR = 8.92 × 10^−9^), and *liver cancer* (FDR = 2.80 × 10^−8^), indicating a strong involvement of liver-related pathological processes. Consistently, *endometrial carcinoma* (FDR = 7.66 × 10^−8^) also showed significant enrichment.

Additional systemic conditions included *ischemia* (FDR = 2.75 × 10^−9^, *respiratory failure* (FDR = 5.22 × 10^−7^), *peritonitis* (FDR = 9.93 × 10^−7^), and *pulmonary edema* (FDR = 9.93 × 10^−7^), suggesting the involvement of processes linked to vascular alterations. Inflammation, and organ dysfunction. In terms of gene representation, *peritonitis* and *pulmonary edema* showed the highest proportions (80%), whereas *endometrial carcinoma* (55.56%), and *liver cancer* (28.57%), also showed high representation within the analyzed gene set.

Human Phenotype Ontology (HPO)-based enrichment analysis identified phenotypes mainly associated with endocrine, reproductive, immunological, metabolic, and neoplastic alterations (Table 4; Figure 6). Among the most significantly enriched phenotypes were *neoplasm by anatomical site* (FDR = 2.7 × 10^−4^), *abnormal immunoglobulin level* (FDR = 1.3 × 10^−3^), and *acanthosis nigricans* (FDR = 2.1 × 10^−3^). The latter finding is noteworthy because of its association with metabolic disturbances, insulin resistance. and endocrine dysfunction.

Phenotypes linked to the endocrine and reproductive systems were also identified, including *polycystic ovaries* (FDR = 3.6 × 10^−3^), *enlarged polycystic ovaries* (FDR = 5.2 × 10^−3^), *macroorchidism* (FDR = 5.2 × 10^−3^), *abnormality of the ovary* (FDR = 2.3 × 10^−3^), and *decreased circulating antibody level* (FDR = 2.5 × 10^−3^), supporting a relevant contribution of hormonal, reproductive, and immunoregulatory processes.

Additional phenotypes such as *breast carcinoma* (FDR = 4.7 × 10^−3^), and *tall stature* (FDR = 2.5 × 10^−3^) further suggest implications related to development. growth. and neoplastic processes.

### 3.3 Upstream transcription factor enrichment analysis

Upstream transcriptional regulator analysis using ChEA3 revealed a recurrent set of transcription factors potentially linked to the glyphosate-associated core gene network. Across both independent runs, seventeen factors were consistently prioritized within the top 20, with average ranking positions ranging from 1.5 to 19 (Supplementary Table S3). Among these, six transcription factors showed the highest consistency and top-ranking positions across both time points: *ZBED6* and *ESR1* (average rank 1.5), *NR5A1* (3.5), *GATA2* and *NR1I2* (6.5), and *SOX7* (European Food Safety Authority (EFSA), 2017), indicating particularly robust regulatory relevance within the analyzed gene set (Table 5).

**TABLE 5:** Upstream transcriptional regulatory landscape of the glyphosate-associated core gene set.

TF	Recurrence	Average rank	Relevant biological evidence	Primary function
*ESR1*	High	1.5	Endocrine disruption	Hormonal regulation
*ZBED6*	High	1.5	Cell proliferation and development	Transcriptional regulation
*NR5A1*	High	3.5	Steroidogenesis and endocrine signaling	Endocrine signaling
*GATA2*	High	6.5	Angiogenesis and differentiation	Vascular development
*NR1I2*	High	6.5	Xenobiotic and nuclear receptor signaling	Xenobiotic metabolism
*SOX7*	High	7.0	Vascular development	Cell differentiation

Regulators were identified using ChEA3 and ranked according to their average rank position across two independent analyses performed with the same gene set. Recurrence across both runs served as an additional criterion of regulatory stability. Lower average rank values indicate higher potential regulatory relevance.

Functionally, the identified regulators converged on the principal biological themes emerging throughout the study. *ESR1, NR5A1, NR1I2*, and *PGR* anchored endocrine and nuclear receptor signaling; *ATF3, EGR1, TP53, JUN,* and *NR4A1* clustered around oxidative stress, inflammation, and apoptosis; while *SOX7, GATA2, ERG, TWIST2, NANOG*, and *HOXD10* reflected roles in cellular differentiation, developmental regulation, and tissue plasticity. *ZBED6* and *ZNF275* contributed broader transcriptional control functions. Despite minor positional variations between runs, the consistent recovery of most factors across independent analyses supports the robustness of the identified regulatory signature. Collectively, these results suggest that the glyphosate-associated gene network operates under interconnected regulatory programs spanning hormonal signaling, stress-response pathways, cellular plasticity, and transcriptional control.

**FIGURE 7:**
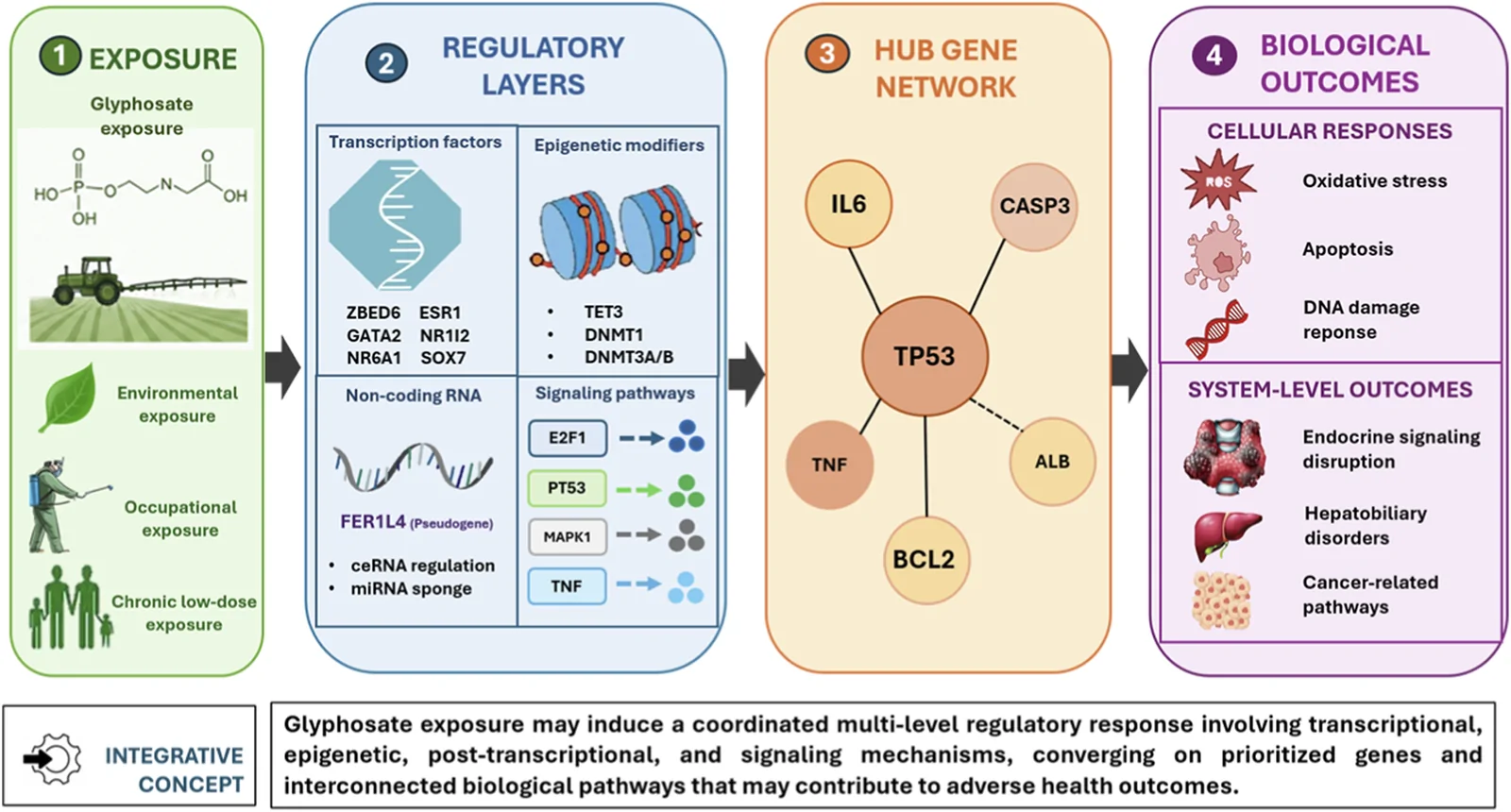
Proposed multi-level mechanistic model underlying glyphosate-associated molecular responses. Schematic integrative model summarizing the regulatory architecture associated with glyphosate exposure. The proposed framework links upstream regulatory elements, including transcription factors, epigenetic modifiers, signaling pathways, and non-coding RNAs, with hub genes identified in the protein–protein interaction network and downstream biological processes related to cellular stress, apoptosis, endocrine signaling, and disease-associated phenotypes.

Functional enrichment, gene-disease association, and Human Phenotype Ontology analyses converged on a consistent biological pattern centered on chemical stress response, apoptosis, inflammation, hormonal signaling, and epigenetic regulation. This pattern was reflected in the overrepresentation of functions related to nuclear receptors, steroid binding, and *DNA methyltransferase activity*, as well as pathways linked to cancer, endocrine resistance, and p53-mediated signaling consistent with the marked representation of hepatobiliary disorders, neoplastic processes, and endocrine and reproductive alterations among the associated phenotypes. Upstream transcription factor analysis further identified *ESR1* and *ZBED6* as the top-ranked regulators, in line with regulatory programs governing hormonal signaling, transcriptional control, and cellular stress response. Together, these findings depict an integrated molecular architecture in which multiple biological processes converge within coordinated regulatory networks associated with glyphosate exposure (Figure 7).

## 4 Discussion

By integrating molecular evidence from multiple complementary sources, this systems biology framework made it possible to move beyond the interpretation of individual genes and biological pathways, revealing three main, interconnected biological axes: (i) cellular stress response and apoptosis regulation, (ii) endocrine signaling and hormone receptor-mediated transcription, and (iii) epigenetic regulation coupled to transcriptional control. The convergence observed across hub genes, functional enrichment analyses, gene–disease associations, human phenotypes, and upstream transcriptional regulators supports the existence of a coordinated molecular network rather than independent biological alterations.

The first and most prominent biological axis emerging from integrative analysis involved cellular stress responses and the preservation of genome integrity. Enrichment of biological processes related to *xenobiotic response*, *reactive oxygen species*, *apoptosis*, and the *p53 signaling pathway* suggests coordinated activation of cellular surveillance mechanisms in response to chemical stress. Consistent with this organization, *TP53, CASP3, BCL2, IL6*, and *TNF* emerged as central regulatory hubs linking apoptotic, inflammatory, and cell fate signaling pathways (Amaral et al., 2010; Aubrey et al., 2018; Gudkov et al., 2011; Mustafa et al., 2024). The highly interconnected protein–protein interaction network further suggests that perturbations affecting a limited number of central regulators may propagate across multiple biological pathways, amplifying their effects at the systems level (Barabási et al., 2011; D'Andrea et al., 2025; Lee and Loscalzo, 2019). Importantly, biological interpretation was based on the convergence of multiple independent analytical layers including functional enrichment, gene-disease and phenotype associations, and upstream transcriptional regulator analyses rather than network topology alone. Accordingly, these hub genes should be interpreted as central regulatory nodes within the prioritized molecular network, rather than glyphosate-specific molecular markers, consistent with network biology models in which hub perturbations coordinate interconnected molecular modules (Barabási et al., 2011; Safari-Alighiarloo et al., 2014). These findings are supported by experimental evidence identifying oxidative stress as a major mechanism underlying glyphosate and glyphosate-based herbicide toxicity, contributing to DNA damage, mitochondrial dysfunction, and programmed cell death (Wang et al., 2022; Martínez et al., 2020; El-Shenawy, 2009).

The biological coherence of this molecular axis was further reinforced by the gene–disease enrichment analysis, which identified hepatobiliary disorders and liver cancer among the most significantly enriched categories. This convergence is biologically plausible given the central role of the liver in xenobiotic metabolism and detoxification (Burcham, 2013; Österreicher and Trauner, 2012), supporting coordinated responses involving oxidative stress, inflammation, apoptosis, hepatocellular injury, tissue remodeling, and tumor progression.

Inflammatory signaling constituted another key component of this molecular axis. The central network position of *IL6* and *TNF*, together with the enrichment of stress- and xenobiotic-related biological processes, suggests that inflammation functions as a regulatory interface linking oxidative stress, apoptosis, and cellular adaptation (Jeong et al., 2019; Karin, 2009; Singh et al., 2019). Beyond immune activation, TNF-mediated signaling contributes to oxidative stress generation, apoptotic regulation, and signaling remodeling, reinforcing inflammation as an integrated component of the molecular response associated with glyphosate exposure rather than an isolated biological process (Jeong et al., 2019; Karin, 2009; Singh et al., 2019).

A second major biological axis involved endocrine signaling and hormone receptor-mediated transcription. Multiple analytical layers converged on this functional theme, including the enrichment of molecular functions related to *steroid binding*, *nuclear receptor activity*, and *estradiol response*, together with the overrepresentation of *endocrine resistance* and *breast cancer pathways*. The identification of *ESR1* as both a central hub within the protein–protein interaction network and an upstream transcriptional regulator further supports the biological coherence of this axis, consistent with the established role of estrogen receptors in regulating cell growth, differentiation, and tissue homeostasis (Green and Carroll, 2007; Li et al., 2020; Nilsson and Gustafsson, 2011). This relevance is particularly evident in breast cancer, where approximately 70% of tumors are estrogen receptor-positive (Lung et al., 2020). Consistent with these findings, experimental studies have shown that glyphosate may interfere with estrogen signaling pathways and exhibit endocrine-disrupting activity under specific experimental conditions (Ingaramo et al., 2016; Chianese et al., 2024).

The concurrent identification of *NR5A1* and *NR1I2* further extends this regulatory network beyond estrogen signaling. *NR5A1* is a key regulator of steroidogenesis and endocrine homeostasis, whereas *NR1I2/PXR* acts as a major xenobiotic sensor controlling the expression of detoxification enzymes and metabolic pathways (Kaur et al., 2025; Mackeh et al., 2018; Reschly and Krasowski, 2006). Together, these findings suggest coordinated interactions among nuclear receptor pathways involved in hormonal regulation, xenobiotic sensing, and transcriptional control, providing additional support for the integrative molecular framework associated with glyphosate exposure (Peng et al., 2025).

The epigenetic axis constituted a third major component of the prioritized molecular network. Enrichment of *DNA (cytosine-5)-methyltransferase activity* suggests the involvement of epigenetic regulation and transcriptional remodeling (Bhootra et al., 2023). Although only a limited number of genes contributed to this enrichment, its high enrichment strength indicates a highly specific functional signal. Given that DNA methylation is a major mechanism through which environmental exposures, including pesticides, regulate gene expression (Larsen et al., 2022; Navarrete-Meneses et al., 2023), these findings are consistent with growing evidence that glyphosate may induce epigenetic alterations, including changes in DNA methylation patterns, with potential long-term effects on gene regulation and disease susceptibility (Kwiatkowska et al., 2017; Woźniak et al., 2021; Ergun and Cayir, 2021; Woźniak et al., 2020).

Upstream transcription factor analysis provided a complementary regulatory perspective for interpreting the prioritized network. The transcription factors identified through ChEA3 converged with the enriched biological processes, particularly those related to hormonal signaling, cellular stress responses, apoptosis, differentiation, and transcriptional regulation. *TP53* and *EGR1* reinforced regulatory programs associated with cellular surveillance, genome integrity, and apoptosis (Aubrey et al., 2018), whereas *SOX7, GATA2*, and *TWIST2* pointed toward coordinated regulation of cellular differentiation, tissue plasticity, and development (Hsu et al., 2015; Shah et al., 2023; Stovall et al., 2013). In contrast, *ZBED6* and *ZNF275* suggest broader transcriptional regulatory programs that remain poorly characterized but are consistent with the regulatory functions of zinc finger transcription factors (Akhtar Ali et al., 2015; Ye et al., 2023). Collectively, these findings support coordinated transcriptional reprogramming across interconnected regulatory networks rather than isolated signaling pathways. Because upstream regulatory inference represents a computational prioritization strategy based on a continuously evolving knowledge base, these transcription factors should be interpreted as candidate upstream regulators prioritized through temporally stable computational inference rather than experimentally validated regulatory drivers and therefore require independent experimental confirmation.

The recurrence of *TP53* across independent analytical layers is particularly noteworthy. In addition to being identified as a central hub within the protein–protein interaction network, *TP53* also emerged as one of the principal upstream transcriptional regulators, reinforcing its role as an integrative node linking oxidative stress, DNA damage responses, cell-cycle regulation, and apoptosis (Shi et al., 2021; Shi and Dansen, 2020). The independent identification of *TP53* through complementary analytical approaches strengthens the biological plausibility of the proposed regulatory framework. Within this context, the coexistence of *TP53* with *TNF, IL6*, and inflammation and apoptosis-related pathways supports coordinated interactions among cellular surveillance, inflammatory signaling, and adaptive stress responses. Notably, TNF represents a central regulatory intersection because it contributes both to caspase-8-mediated apoptotic signaling (Gupta et al., 2025) and to inflammatory and pro-survival responses through *NF-κB* activation (Singh et al., 2019; Bazhanova and Kozlov, 2026). Together, these findings support an integrated molecular network in which multiple adaptive regulatory processes respond coordinately to glyphosate-associated chemical stress rather than acting as independent biological alterations (Karin, 2009; Singh et al., 2019; DiDonato et al., 2012).

Beyond transcriptional regulation, the incorporation of *FER1L4,* a pseudogene functioning as a long non-coding RNA (lncRNA), adds a post-transcriptional regulatory layer to the proposed network. The integration of *FER1L4*-associated genes suggests the involvement of ceRNA-like mechanisms capable of modulating genes related to apoptosis, cellular stress responses, and proliferative control (Alkan and Akgül, 2021; Gao et al., 2019; Ma et al., 2019). This observation further supports a multilayered regulatory architecture integrating epigenetic, transcriptional, and post-transcriptional mechanisms.

Taken together, these findings demonstrate that integrating complementary molecular evidence through a reproducible systems biology workflow enables reconstruction of a coordinated regulatory architecture associated with glyphosate exposure. Rather than representing isolated molecular alterations, the identified biological axes reveal interconnected regulatory layers involving cellular stress responses, endocrine signaling, epigenetic regulation, transcriptional control, and post-transcriptional modulation. The convergence across protein-protein interaction networks, functional enrichment, gene-disease associations, human phenotypes, and upstream regulatory analyses strengthens the biological coherence of this framework and supports molecular prioritization for hypothesis generation, biomarker discovery, and future mechanistic and epidemiological studies.

A major strength of this study is the systematic integration of complementary molecular evidence from curated toxicogenomic databases, literature-derived resources, AI-assisted information retrieval, and independent transcriptomic datasets within a unified analytical workflow. Using predefined selection criteria and multiple complementary analytical layers including protein-protein interaction networks, functional enrichment, disease and phenotype associations, and upstream transcriptional regulator analysis the workflow prioritized candidate genes consistently supported across independent sources. This integrative strategy reduced reliance on isolated observations and provided a biologically coherent framework for interpreting the molecular responses associated with glyphosate exposure.

## 5 Limitations

This study has limitations inherent to its integrative systems biology approach, which is based on secondary data sources and *in silico* inference and therefore does not allow causal interpretation of the identified molecular associations. Although AI-assisted gene retrieval was subjected to manual curation and corroboration against independent databases, potential selection bias cannot be completely excluded, and the quality of the results ultimately depends on the completeness and reliability of the underlying databases. In addition, the integration of heterogeneous experimental models, together with the absence of quantitative exposure information and direct experimental validation, limits the immediate biological interpretation of the proposed molecular framework and precludes its application to toxicological risk assessment. Consequently, the prioritized molecular candidates and regulatory networks generated by this workflow should be regarded as hypothesis-generating resources requiring further validation through controlled experimental models and well-characterized epidemiological studies.

A further limitation concerns the ChEA3-based transcription factor analysis: as a continuously updated resource, ChEA3 offers no version control or archival access, so regulatory rankings may shift over time regardless of the underlying biology. To mitigate this, prioritization was assessed at two time points 3 weeks apart, retaining only factors consistently recovered in both. This provides empirical evidence of relative temporal stability, but not formal validation of regulatory relevance, which would require experimental confirmation.

Finally, although the specificity of the identified hub genes to glyphosate exposure was established upstream, through convergence across independent sources rather than network topology itself, we did not benchmark network centrality against randomly selected or unrelated-exposure gene sets. Such comparisons would help distinguish biologically meaningful hubs from generic, highly connected nodes common to biological networks, and represent a valuable direction for future validation.

## 6 Conclusions

This study developed and applied a reproducible integrative systems biology workflow that combined curated databases, transcriptomic evidence, artificial intelligence-assisted retrieval, network-based analyses, and upstream regulatory analyses to prioritize a set of 50 glyphosate-associated genes. Rather than pointing to isolated molecular alterations, the integrated analyses reconstructed a coordinated molecular architecture characterized by interconnected regulatory processes involving cellular stress responses, apoptosis, inflammation, endocrine signaling, epigenetic regulation, and tissue remodeling.

Collectively, these findings demonstrate the value of integrating complementary molecular evidence to reconstruct a systems-level molecular architecture of glyphosate-associated biological responses. The proposed framework provides a reproducible strategy for molecular prioritization and hypothesis generation, offering biologically grounded molecular candidates and regulatory networks to guide future mechanistic, experimental, and epidemiological studies. It should be emphasized that the proposed framework is intended for molecular prioritization and hypothesis generation, rather than for causal inference or toxicological risk assessment.

## Data Availability

The raw data supporting the conclusions of this article will be made available by the authors, without undue reservation.
