# Correction: Network-based integrative analysis of multi-level regulatory mechanisms associated with glyphosate exposure

**DOI:** 10.3389/fbinf.2026.1979796

**Published:** 2026-09-09

**Authors:** Claudia Consuegra-Mayor, Barbara Arroyo-Salgado, Jesus Olivero-Verbel

**Affiliations:** 1 Environmental and Computational Chemistry Group, School of Pharmaceutical Sciences, Zaragocilla Campus, University of Cartagena, Cartagena, Colombia; 2 Biomedical, Toxicological and Environmental Sciences Group, School of Medicine, Zaragocilla Campus, University of Cartagena, Cartagena, Colombia; 3 School of Health Sciences, University of San Buenaventura, Cartagena, Colombia

**Keywords:** biomarkers, epigenetic regulation, hub genes, network biology, transcriptional regulation

In the funding section, the correct Funding statement should be: “The author(s) declared that financial support was received for this work and/or its publication. This study was funded by the Colombian Ministry of Science, Technology, and Innovation (Minciencias, grant 1107103390097) and by the Office of the Vice Rector for Research at the University of Cartagena, Commitment Agreement 041-2025.”

The original version of this article has been updated.

